# Subjective energy poverty and attitudes on climate change mitigation measures: Empirical and ethical considerations

**DOI:** 10.1007/s13280-026-02434-7

**Published:** 2026-06-28

**Authors:** Jukka Sivonen, Helena Siipi, Vuokko Härmä, Sakari Karvonen

**Affiliations:** 1 Finnish Institute for Health and Welfare, P.O. Box 30, 00271 Helsinki, Finland; 2 University of Turku, Assistentinkatu 7, 20500 Turku, Finland

**Keywords:** Climate nudge, Climate policy, Energy vulnerability, Public opinion, Public perceptions, Survey research

## Abstract

Objective energy poverty—commonly defined using indicators such as the relationship between energy costs, income, and energy required for adequate living—has been widely studied, yet less is known about how subjective energy poverty shapes attitudes toward climate change mitigation measures. This study examines theoretical and empirical links between subjective energy poverty and support for climate measures, including fossil fuel taxation and two climate nudges. We argue that “leaving behind” harm arises from subjective energy poverty independently of objective conditions, whereas economic harms from climate nudges are more likely among individuals experiencing only objective energy poverty. Empirically, we analyze nationally representative Finnish survey data from 2023 (*n* = 5909) using ordered probit regression. Results show that higher subjective energy poverty is associated with lower support for climate measures. The findings highlight the importance of addressing energy poverty in designing and communicating climate policy.

**Supplementary Information:**

The online version contains supplementary material available at 10.1007/s13280-026-02434-7.

## Introduction

In an ideal situation, we could recognize climate change mitigation measures, or their combinations, that are effective, ethically good and widely supported. However, effective measures that significantly decrease emissions often lack wide public support (Drews and van den Bergh 2015). Therefore, it is important to better understand citizens’ attitudes toward different climate mitigation measures and the factors associated with these attitudes. This study focuses on energy poverty as one such factor related to public support for climate policies.

Energy poverty has received growing attention in both research and public debate. It can be approached from objective and subjective perspectives, both capturing different forms of deprivation. Objective energy poverty typically refers to measurable relationships between energy costs and income, such as the widely used threshold of spending more than 10% of disposable income on energy (Boardman 1991). More recent approaches estimate the energy required to reach basic comfort levels while accounting for household characteristics and geographic conditions (Faiella and Lavecchia 2021) and may also incorporate cooling needs (Gouveia et al. 2019). Energy poverty has been shown to affect health, well-being, social inclusion, and carbon reduction efforts (Hills 2012).

Recent empirical work further highlights its multidimensional nature. Some households deliberately underconsume energy due to financial pressure, masking hardship in expenditure-based metrics (Eisfeld and Seebauer 2022). Community-level studies link energy poverty to poorer physical and mental health and increased financial stress (Riva et al. 2024). Comparative analyses across EU countries show that alleviation is most effective when policies simultaneously address income, housing energy performance, and energy prices (Kyprianou et al. 2019). Energy poverty is therefore a structural issue that cannot be resolved solely through individual choices (Middlemiss and Gillard 2015). Reflecting this complexity, Castaño-Rosa et al. (2019) classify indicators into income/expenditure measures, self-reported housing conditions, and risk-based assessments, while Charlier and Legendre (2019) propose a non-binary index capturing monetary constraints, poor energy efficiency, and heating deprivation.

Subjective energy poverty refers to individuals’ own perceptions of their inability to afford sufficient energy to meet their basic needs. It is typically measured through surveys in which respondents assess their situation across several dimensions. Aristondo and Onaindia (2018) highlight experienced difficulties maintaining adequate warmth, structural housing problems such as dampness or roof leaks, unpaid energy bills, and inefficiencies caused by poor insulation as key indicators. Other scholars emphasize additional aspects: Middha and Willand (2023) describe energy insecurity as emerging when households must choose between energy and other necessities such as food, experience anxiety about potential disconnection, or cope with inadequate appliances or impractical tariff systems. Because energy use is embedded in many everyday practices beyond heating, such as cooking and other domestic routines, the experience of energy poverty is shaped by complex, multidimensional interactions across daily life (Middha and Willand 2023).

While energy poverty is often considered a static or chronic condition, both objective and subjective energy poverty are also linked to temporal and situational dimensions (Burlinson et al. 2021; Bridgen and Robinson 2023). Objective energy poverty may fluctuate in response to external factors such as rising energy prices, economic downturns, or unemployment. Subjective energy poverty, by contrast, is particularly sensitive to temporal uncertainty and the stress it generates.

Although subjective energy poverty is often influenced by the same structural conditions as objective energy poverty, it also encompasses worries and anxieties, actual or anticipated, about one’s ability to cope with financial hardship arising from energy costs. Moreover, an unexpected, even if one-off, increase in an electricity bill can have long-term financial consequences for a low-income household, weakening its future economic resilience. From a service-based perspective, energy poverty can be understood as the inability to attain necessary domestic energy services, regardless of whether this stems from income, energy prices, or housing conditions (Bouzarovski and Petrova 2015), highlighting how both immediate shocks and longer-term constraints shape lived experiences of energy deprivation.

Subjective energy poverty is critically contextual in nature, as for instance the ideal indoor temperatures and the associated costs can vary widely across different locations and cultures (Teixeira et al. 2024). Furthermore, changes in climate and extreme weather events may heighten anxieties about affordability and reliability of energy services. Such variability is crucial to consider when designing and implementing climate change mitigation measures. In practice, there are often trade-offs and competition points between the agendas of climate change mitigation and energy poverty alleviation (Mahoney et al. 2024) so that climate measures may inadvertently escalate objective energy poverty (Belaïd 2022; Belaid et al. 2023; Vandyck et al. 2023). As many climate mitigation measures imply increases in energy costs, they are likely to influence subjective energy poverty. In the Finnish context, the cold climate makes access to affordable and reliable heating essential, as heating expenses account for a significant share of household budgets; in 2021, 18% of households spent over 10% of their disposable income on energy, 27% considered energy costs a regular financial burden, and 8% faced both conditions simultaneously (Numminen et al. 2024). Furthermore, 2.7% of Finns reported being unable to keep their homes adequately warm in 2023, the year in which our data were collected (Eurostat 2026).

Subjective energy poverty captures lived experiences, including stress and difficulty meeting energy needs. Because of this, it can also predict attitudes toward public policies. Subjective energy poverty can lead to significant societal effects, including shaping behaviors and attitudes toward climate change mitigation (Sarafidis et al. 2024). Thus, an understanding of subjective energy poverty is crucial for comprehensively addressing its broader impacts. Despite extensive research on the effects of climate policies on energy poverty, there remains a significant gap in understanding how subjective energy poverty influences public attitudes toward climate policies. An exception is a study indicating that in Greece, those experiencing energy poverty are less willing to pay for climate change mitigation measures (Sarafidis et al. 2024).

Our study also discusses the ethical considerations, focusing on the potential financial, psychological and social burden that climate change mitigation measures can cause and unites them with subjective and objective energy poverty. This approach does not take a normative stand on moral questions. Rather it integrates theoretical considerations with empirical research to propose more effective communication and implementation strategies for climate policies that account for the diverse lived experiences of energy poverty. Moreover, as noted earlier, there is an empirical research gap on the relationship between experienced energy poverty and attitudes on climate change mitigation measures. This study aims to bridge this gap by studying the associations between subjective energy poverty and attitudes on three measures: fossil fuel taxation and the following two climate nudges: the first concerning making renewable electricity the default in electricity contracts, and the second about displaying low-emission products in the most accessible locations in stores.

To explore these themes, our research questions are:

RQ1: From a theoretical perspective, is objective and/or subjective energy poverty a risk factor for ethically noteworthy harms from climate nudges?

RQ2: From an empirical perspective, how do public attitudes vary between different climate change mitigation measures?

RQ3: How is subjective energy poverty associated with support for different climate change mitigation measures?

## Subjective and objective energy poverty and ethically noteworthy harms

Earlier studies have identified risks of ethically noteworthy harms regarding climate change mitigation measures that bring about non-avoidable increases in energy costs. In particular, high fossil fuel taxes burden those low-income groups who are dependent on fossil fuels for their daily practices such as heating their homes (Cottrell and Falcão 2018; Ghesla et al. 2020; Mahoney 2024; Romero et al. 2025). The two climate nudges under interest in this study can also bring about increases in energy costs. On climate nudges, however, the costs can be avoided by active choice. In what follows, we will theoretically argue for the following claims: First, climate nudges can bring about ethically noteworthy harms of *leaving behind* and *harming outright*. Second, the ethically noteworthy harm of *leaving behind* arises from subjective energy poverty and does not depend on objective energy poverty. Third, *harming outright* is especially likely regarding those who are in objective, but not in subjective, energy poverty.

As noted above, we will discuss the following two climate nudges: first, making renewable electricity the default in electricity contracts, and second, displaying low-emission products in the most accessible locations in stores. As long as renewable energy and low-emission products are more expensive than their alternatives, these climate nudges cause economic costs to those consumers who follow them. The costs can be avoided by not following the default and by choosing less easily accessible products. Even though increase in energy costs is formally avoidable, these measures nonetheless require critical scrutiny. We argue that such nudges may have a disproportionate impact on those experiencing energy poverty, as they can *leave them behind* or *harm them outright* (Roberts 2018; Thunström et al. 2018). We first present these two ethically noteworthy harms of nudges and then apply them to objective and subjective energy poverty.

Some nudges are ethically problematic because they *leave behind* those who do not have resources and capacities to follow them. For instance, a group health challenge that is based on social norms around physical activity may fail in contexts where structural barriers such as lack of transportation, irregular work schedules, or caregiving responsibilities make it difficult for individuals to commit to this exercise. In such cases, the health-nudge not only becomes ineffective but also generates ethically noteworthy harms: social comparison to others, internalized inadequacy and heightened stress from an inability to fulfill to the normative expectations (Roberts 2018; Fowler and Roberts 2019; Siipi and Koi 2022). Similar dynamics take place regarding climate nudges. Displaying low-emission products in the most accessible locations creates a social norm to favor them. Ethically noteworthy harms—comparison, internalized inadequacy and stress—fall on those who for economic reasons have to choose the less climate wise alternatives. Energy poverty then harms individuals by hindering their ability to freely decide how to engage in climate actions (Della Valle et al. 2024).

Second, some climate nudges *harm vulnerable targets outright* (Roberts 2018). Research suggests that groups with lower levels of education or income are more likely to adhere to default options—even when doing so causes higher personal costs (Sunstein 2016). The capacity to critically assess behavioral interventions is not the same for everyone. Situational factors, including scarcity, cause cognitive load which limits possibilities for contemplating over different alternatives and making optimal choices (Della Valle 2019; Siipi and Koi 2022). The context of scarcity makes people more likely to follow defaults, not out of preference but the limited possibilities to opt out. This implies, that defaults for renewable electricity, may economically disadvantage people:

Suppose, for example, that the cost-benefit analysis argues in favor of a green default, but that the selection of that default imposes net costs on consumers, including poor people. Suppose too that poor people are unlikely to opt out, perhaps because they are busy and occupied with other matters, perhaps because they are not confident that opting out makes best sense. If poor people would in fact be net losers but would not opt out, the argument for a green default is weakened. (Sunstein 2016, 179. See also Sunstein 2017, 5)

Nudges such as defaults for renewable electricity or strategic placements of low-emission products in stores reproduce similar dynamics. Renewable energy and low-emission products are proportionally more expensive to those who have less funds. Those with less funds are more likely to follow the nudges and more likely to suffer from the economic costs the defaults bring about. To sum up, when climate nudges are built without taking into account the differences in people’s economic situation, they can lead to *leaving behind* and *harming outright* those who are lacking resources (for further discussion see Romero et al. 2025).

The ethically noteworthy harms of *leaving behind* and *harming outright* become even more likely and severe when studied from the viewpoints of subjective and objective energy poverty. This is due to the fact that subjective and objective energy poverty do not always go together. Rather, we have four possible cases (a, b, c and d; see Table 1).

**Table 1:** Cross-tabulation of objective and subjective energy poverty

	Subjective energy poverty	No subjective energy poverty
Objective energy poverty	a	c
No objective energy poverty	b	d

For the sake of the argument, we suppose that the costs of the climate nudges (default for green energy and displaying low-emission products in the most accessible locations) are such that the people who are not in energy poverty do not suffer significantly from them. In the situation of energy poverty the defaults are taken to bring about economic losses that may compromise access to basic goods (such as nutrition, health care, clothing and transport).

The case d then covers optimal targets of the renewable electricity defaults and low-emission product displays. They can afford to follow the nudge, and they themselves judge that they can afford it (even though following it may compromise access to some luxuries).

Case a comes close to what is above described as *leaving behind* harm. In case a, the economic costs of the green default are so high that followers would suffer ethically noteworthy economic harms. The subjects acknowledge this and choose not to follow the default (Sunstein 2017). As noted above, not following it may bring about social or psychological ethically noteworthy harms to the subjects.

The case b is an ethically noteworthy instance of *leaving behind* harm. In case b, the subjects are not in objective energy poverty, but they perceive themselves as suffering energy poverty. This kind of subjective energy poverty is sufficient for making the subjects *nudge-proof* (just like individuals of case a). They opt-out due to their self-judgment that they cannot afford green energy or low-emission products. Not following the nudge leads to exactly the same social and psychological harms as in the case a.

Cases a and b are similar regarding the possible *leaving behind* harms to the subjects. Yet, they differ with respect to their societal outcomes. Case b implies losing some possibilities for decarbonization and climate benefits. The targets opt out, even though they, objectively speaking, should not.

Case c is an ethically noteworthy instance of *harming outright*. The defaults for green energy and making low-emission products most easily available harm their targets outright (for the reasons described earlier) when people in objective energy poverty realize their economic situation (case a). Following the defaults or choosing easily available items is even more likely when people in objective energy poverty do not suffer subjective energy poverty—that is when subjects do not realize their objective energy poverty (case c). In both cases following, the nudge can cause significant economic burden.

The problem in case c is that people do not opt-out even though they (for the sake of their own well-being) should. So-called spendthrifts—people who have a tendency to overspend—may be especially vulnerable to these nudges (Thunström et al. 2018). Defaults for green energy and easy access to low-carbon products may lead them into economic situations in which they have difficulties fulfilling their basic needs. Thus, the case c calls for ethical attention.

The distinction between objective and subjective energy poverty—together with Roberts’s analysis of the ethically noteworthy harms of nudges—highlights how a single nudge may be ethically problematic in both being over-inclusive (affecting people who should for the sake of their own well-being opt-out) and under-inclusive (not affecting people who for the sake of the climate should opt-in). The former is especially likely to take place when targets are in the objective but not in subjective energy poverty (case c). The latter is likely when people suffer subjective energy poverty without objective energy poverty (case b). These risks of ethically noteworthy harms are a previously unnoticed instance of trade-offs between climate measures and energy poverty agendas. Taken together, the above analysis shows the importance of taking the distinction between subjective and objective energy poverty into account in decision-making over different climate change mitigation measures including climate nudges.

The above analysis shows that both subjective and objective energy poverty are risk factors for ethically noteworthy harms from climate nudges. These risks may partly explain negative attitudes toward climate nudges. In what follows, we compare the measures of renewable energy defaults, easily available low-carbon products and fossil fuel taxes with respect to people’s willingness to support them. We analyze whether the supportive attitudes regarding different measures are in line with the possible ethically noteworthy harms spelled out above.

## Climate change mitigation measures and public attitudes

Although there is broad scientific consensus on the need to mitigate climate change, there are many possible ways to achieve this goal, and experts and laypeople often disagree on which measures are most appropriate. Effective climate policy typically requires a mix of instruments rather than a single solution (Sterner and Robinson 2018).

This study examines three mitigation measures: fossil fuel taxation, setting renewable electricity as the default option in electricity contracts, and placing low-emission products in the most accessible locations in stores. The first is a price-based measure, while the latter two are climate nudges discussed earlier from an ethical perspective. Price-based instruments aim to reduce emissions by increasing the cost of carbon-intensive actions or lowering the cost of climate-friendly alternatives (Sterner and Robinson 2018). Fossil fuel taxes exemplify the former. Although climate nudges may also increase electricity costs, individuals can avoid these costs by opting out. In Finland, nudges generally enjoy relatively high public support (Sivonen et al. 2025), and also fossil fuel taxation—typically unpopular across countries—has received comparatively high support (Pohjolainen et al. 2018).

Fossil fuel taxes can encourage more climate-friendly consumption and investment and may stimulate climate-beneficial innovations (Hsu 2016). They are widely recognized as cost-effective tools for reducing emissions (Sterner and Robinson 2018). However, they tend to be unpopular (Pohjolainen et al. 2018), partly because households perceive short-term economic risks (Povitkina et al. 2021). Without compensatory measures, carbon taxes may also exacerbate social inequalities and, over time, shrink their own tax base as fossil fuel use declines. Those in subjective energy poverty are particularly unlikely to support such taxes, as they risk increasing financial strain.

Setting renewable electricity as the default option in electricity contracts represents a classic low-carbon nudge that leverages people’s tendency to stick with default choices (Schubert 2017). Studies across Europe show high approval for this type of nudge (Miłaszewicz 2022), and evidence from Germany indicates substantial effectiveness, with green energy purchases increasing tenfold (Ebeling and Lotz 2015). However, green contracts may be more expensive, potentially burdening low-income households (Ghesla et al. 2020). Subjective energy poverty is expected to decrease support for this type of nudge, as it involves the risks of higher energy bills and of the ethically noteworthy harms spelled out in the last section of this paper.

Displaying low-emission products in the most accessible locations in stores also utilizes elements of default green nudge type that utilize people’s tendency to stick to assumptions and avoid active choice (Schubert 2017). In the context of a cafeteria, this sort of nudge was highly supported in the US and Sweden (Hagman et al. 2015). However, products with lower emissions are usually more expensive than other products. Thus, it can be assumed that such product placement includes a risk of increasing the costs if customers choose low-emission options due to their prominent placement. As noted in the theoretical analysis, such risk is especially likely when people are in objective but not in subjective energy poverty. Hence, experiencing energy poverty could reduce support for this kind of nudge. However, we can assume that product placement in stores is easier to avoid than offering a specific electricity contract as the default option. Therefore, the coercive effect is presumably smallest in the case of product placement among these three climate measures.

As noted earlier, empirical research on how subjective energy poverty shapes attitudes toward climate mitigation measures remains limited, even though subjective energy poverty likely influences perceptions of costs, fairness, and acceptability. Climate mitigation and energy poverty alleviation may also involve conflicting goals, as some mitigation policies can increase household energy costs (Mahoney et al. 2024). This study addresses this gap by examining how subjective energy poverty relates to attitudes toward fossil fuel taxation and two climate nudges. The next section presents the data and methodology used in the empirical analysis.

## Data and methodology

To address our research questions, we use data from the Climate Nudge Survey, collected in Finland between May and August 2023. A random sample of 20 000 individuals aged 20–85 was drawn from the Finnish Population Register, and 5909 respondents completed the survey (response rate 30%). The questionnaire was available in Finnish and Swedish (paper and online) and in English (online only). Table 2 summarizes the data.

**Table 2:** Details of the climate nudge-survey data

Item	Details
Dataset	Climate Nudge-survey
Country & period	Finland; May–August 2023
Target population	Finns aged 20–85
Sampling	Random sample from population register
Sample/responses	20 000 invited; 5909 completed
Response rate	30%
Survey mode & languages	Paper & online (Finnish, Swedish); English online only

The questionnaire inquired respondents’ attitudes on different climate change mitigation measures. Following questions were included in this study:

- Increasing taxation of fossil fuels, such as oil, natural gas and coal
- Making renewable electricity the default option for electricity contracts, even if it is slightly more expensive than other forms of energy
- Displaying low-emission products in the most accessible locations in stores

The response scale was: (1) “I would strongly support it,” (2) “I would somewhat support it,” (3) “I would neither support or oppose it,” (4) “I would somewhat oppose it,” and (5) “I would strongly oppose it.” To enable more intuitive interpretation, in the regression analysis the original scale was reversed so that 1 = ”I would strongly oppose it” and 5 = ”I would strongly support it.” Descriptive statistics of the dependent variables can be seen in Table S1.

Our independent variable, subjective energy poverty, was asked with the question “I have to compromise on my basic needs in order to pay my energy bills.” The definition of energy poverty used in our analysis was adopted from Runsten et al. (2015). Response scale was: (1) “Completely agree,” (2) “Fairly agree,” (3) “Neither disagree or agree,” (4) “Fairly disagree,” (5) “Completely disagree.” In the regression analysis, the scale was reversed to make interpretation more intuitive.

In the following, we present our control variables. Gender was self-reported with four options: (1) “Woman” (2) “Man” (3) “Other” and (4) “I do not want to tell. Due to few observations in other categories, only 1 and 2 were included in the analysis. The subjective evaluation of the respondent’s own financial situation was inquired as follows: “How would you evaluate your economic situation at the moment?” with answer options (1) “Very good,” (2) “Quite good,” (3) “Moderate,” (4) “Quite poor,” (5) “Very poor.” To ensure that enough respondents are represented in each category and to make better readable results, the variable was recategorized and reversed so that the final categories were (1) “Bad,” (2) “Moderate,” and (3) “Good.”

Objective income was measured using five brackets of annual household income before taxes. To account for household size, income was equivalized using the OECD (2023) scale (1.0 for the first adult, 0.5 for additional adults and children aged 7 +, and 0.3 for children under 7). Household composition was derived from the survey. Income was log-transformed to reflect proportional differences across income levels (Ermini and Hendry 2008).

Education was reported as the highest completed degree and recoded into three categories: primary, secondary, and tertiary. Age was calculated from birth year and categorized into three groups: 20–41, 42–63, and 64–85 (mean 50.72; SD 17.87). Descriptive statistics for all independent variables appear in Table S2.

Party preference was measured by asking respondents how they would vote if parliamentary elections were held at the time of the survey. All parliamentary parties were listed. Smaller parties (Swedish People’s Party, Christian Democrats, Movement Now) were combined into an “Other” category. “Other party” and “Would not vote” were included in the models but omitted from result tables for clarity. In the Finnish political landscape, the Left Alliance is left-of-center-left, the Social Democrats center-left, the Green League green center-left, the Centre Party rural-centrist, the National Coalition Party center-right, and the Finns Party populist right (Saarinen et al. 2018).

These sociodemographic and political variables were included because prior research shows they influence climate policy attitudes (Davidovic and Harring 2020; Sivonen et al. 2025). All variables were tested for multicollinearity; variance inflation factors were below the commonly accepted threshold of 5 (Yu et al. 2015).

Because the dependent variables are ordinal, we use ordered probit regression. This method estimates how predictors influence the probability of responses across ordered categories. Positive coefficients indicate a higher likelihood of supporting the measure, while negative coefficients indicate lower likelihood of support (Daykin and Moffatt 2002). Cut points represent the thresholds between observed response categories.

To correct for age and gender biases, analyses were weighted using population data from Statistics Finland (2025). All analyses were conducted in Stata 17.

## Results

**Fig. 1:**
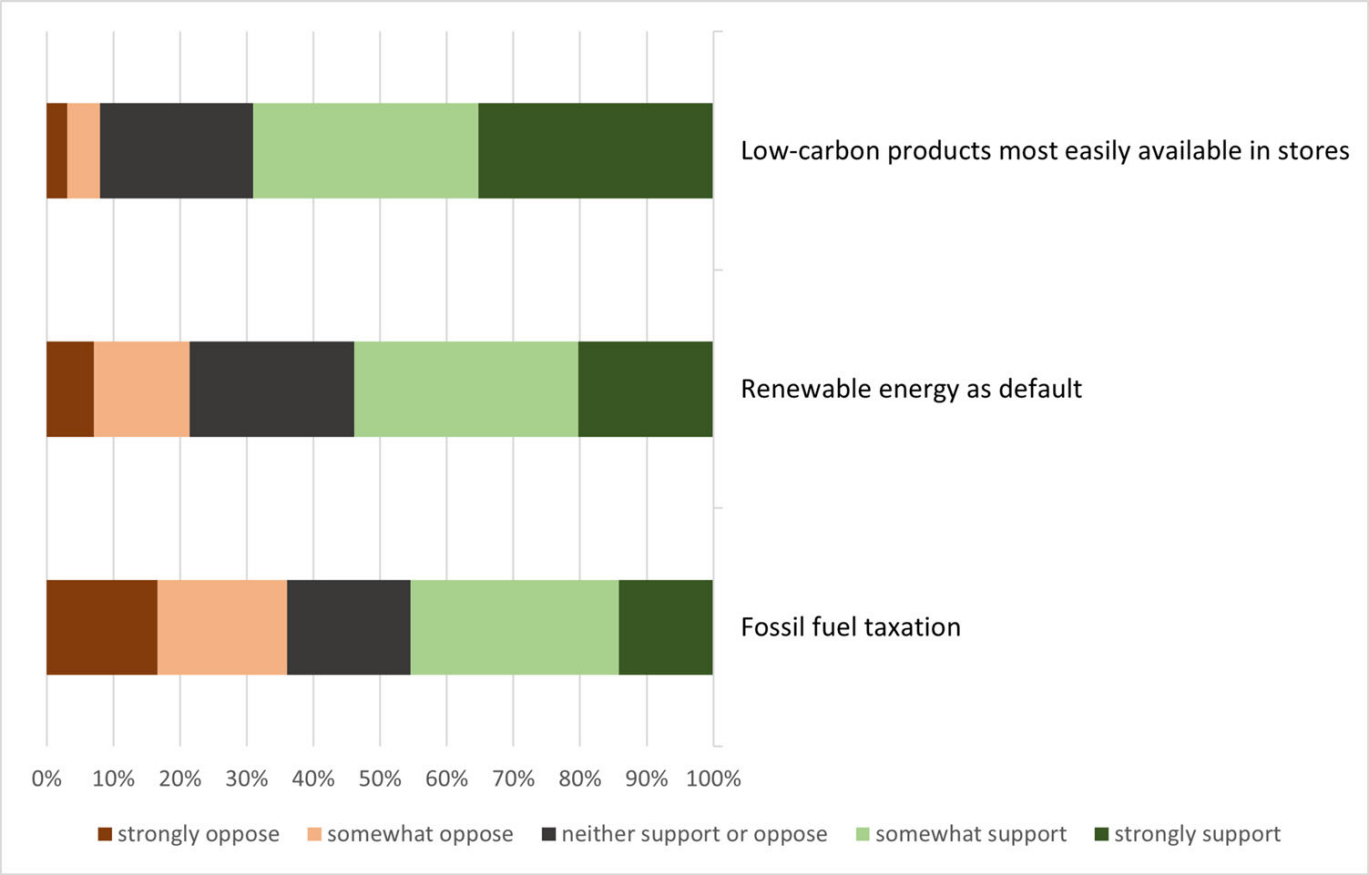
Distributions (%) of attitudes on different climate change mitigation measures. Number of respondents: fossil fuel taxation = 5576, renewable energy as default = 5568, and low-carbon products most easily available in store = 5560

Figure 1 shows that making low-carbon products more accessible in stores is supported by 69.0% of respondents, indicating a strong preference for having sustainable options readily available. Over half, at 53.9%, support setting renewable energy as the default option in energy when making electricity contracts. Support for a higher fossil fuel taxation stands at 45.4%, showing a more reserved but notable approval for fiscal measures aimed at reducing carbon emissions. More detailed information about the climate change mitigation measure-variables and their distributions can be seen in Table S1. When it comes to subjective energy poverty, 27.5% of respondents reported agreeing at least slightly with the statement that they must compromise on meeting their basic needs due to energy costs (Table S2).

Table 3 presents the findings from ordered probit regressions that assess the association between subjective energy poverty and attitudes toward three different climate change mitigation measures: support for increasing fossil fuel taxation, offering renewable electricity as a default, and making low-carbon products the most accessible.

**Table 3:** Ordered probit regressions on the associations between subjective energy poverty and attitudes towards different climate change mitigation measures. M1 does not include control variables and M2 includes sociodemographic control variables. Coefficients (robust standard errors in parentheses). *p < 0.05, **p < 0.01, ***p < 0.001

Increasing fossil fuel taxation	M1: Without controls	M2: With controls
Subjective energy poverty	− 0.193*** (0.0142)	− 0.118*** (0.0153)
N	4450	4450
Pseudo R2	0.0414	0.1228

Offering renewable electricity as default	M1: Without controls	M2: With controls
Subjective energy poverty	− 0.152*** (0.0140)	− 0.085*** (0.0151)
N	4452	4452
Pseudo R2	0.0124	0.0825

Putting low-carbon products as most accessible	M1: Without controls	M2: With controls
Subjective energy poverty	− 0.0986*** (0.0142)	− 0.0685* (0.0152)
N	4455	4455
Pseudo R2	0.00560	0.0738

For each climate change mitigation measure, the analysis is split into two models: Model 1 (M1), which does not include sociodemographic controls, and Model 2 (M2), which does. The results showed that higher subjective energy poverty was associated with lower support for all three dependent variables. These findings remained statistically significant when the control variables were added to the analysis in M2. Information about the associations between control variables and dependent variables is not included in Table 3, but these can be seen in Appendix Table S3.

The explanatory power of the models, as indicated by the pseudo-R2 values, differed across the climate change mitigation measures. Among the measures examined in M1, the model for fossil fuel taxation showed the highest pseudo-R2 value, indicating that the predictors included in the model collectively improve the model fit more for this outcome than for the other policy measures. In contrast, the models explaining attitudes toward renewable electricity as the default option and the placement of low-carbon products as the most accessible alternative showed lower pseudo-R2 values, suggesting a comparatively weaker overall model fit for these outcomes.

When sociodemographic and political control variables were added in M2, the pseudo-R2 values increased for both climate nudge measures, whereas the increase was smaller for attitudes toward fossil fuel taxation. This indicates that adding these control variables improves the overall model fit more strongly for the nudge policies than for fossil fuel taxation. Thus, while subjective energy poverty remains statistically significantly associated with attitudes toward both nudges, the improvement in model fit after adding controls suggests that sociodemographic characteristics and party preferences contribute more to explaining variation in attitudes toward default renewable electricity contracts and product placement nudges than toward fossil fuel taxation.

To summarize, fossil fuel taxation was the least supported climate measure, while making low-emission products the most accessible in stores was the most supported. Higher experiences of energy poverty were more strongly associated with lower support for fossil fuel taxation than with the two types of climate nudges. Nevertheless, all these associations remained statistically significant when control variables were included in the models.

## Discussion and conclusions

This study analyzed Finns’ attitudes on different climate change mitigation measures and how subjective energy poverty is associated with those attitudes both from a theoretical and an empirical point of view. Higher subjective experience of energy poverty was linked to lower support for all the climate measures we examined: fossil fuel taxation, offering green electricity contracts as default, and putting low-emission products most easily available in stores. Therefore, the experience of energy poverty appears to be a relevant factor in the design of climate change mitigation strategies and curbing it could help to increase public support for climate policy.

The two main conclusions of the theoretical analysis can be stated as follows. First, not only climate change mitigation measures that bring about non-avoidable increases in energy costs, but also measures from which people can opt out, may disadvantage people. Second, the distinction between objective and subjective energy poverty enables recognizing previously unnoticed risks of ethically noteworthy harms from climate nudges. The *leaving behind harm* may fall on those who are both in objective and subjective energy poverty but it also concerns those who suffer only subjective energy poverty. The risk of *harming outright* is especially severe regarding people who are only in objective energy poverty, but not in subjective energy poverty. Thus, as a response to our RQ1, both mere subjective energy poverty and mere objective energy poverty are risk factors for ethically noteworthy harms from climate nudges. The distinction, thus, should be recognized in decision-making regarding climate change mitigation measures. Moreover, the risk of ethically noteworthy harms offers one possible explanation for the results of the empirical analysis. In short, lower support may follow from the possibility of these harms. People who are experiencing subjective energy poverty may, because of their situation, be especially good at spotting the risk factors of different climate measures. Moreover, they may see themselves as potential sufferers of these harms. The apprehension of the risks may lead to lesser support.

Potential harms of different climate change mitigation measures are relevant to decision-making concerning them. However, that a measure may cause some harm does not imply that it should never be carried out. Rather measure’s harms should be weighed against its benefits as well as compared to potential harms and benefits of alternative measures (Mahoney et al. 2024). Among other things, people’s attitudes regarding different measures should be acknowledged in decision-making. Moreover, their opinions should be seen as embedded in their lived experiences.

Public attitudes toward the three mitigation measures differ clearly, addressing RQ2: How do public attitudes vary between different climate change mitigation measures? Descriptive results show that placing low-carbon products in the most accessible store locations received the highest support among Finns, whereas fossil fuel taxation was the least supported. Fossil fuel taxation represents a more coercive policy instrument, while the two nudges examined in this study allow individuals to opt out of potential additional costs. It is also reasonable to assume that avoiding higher costs resulting from product placement is easier than opting out of a default electricity contract, making the coercive impact of product placement the weakest of the three. Prior research indicates that more coercive climate policies tend to receive lower public support than less coercive ones (Drews and van den Bergh 2015), and our findings align with earlier studies on Finnish attitudes toward climate measures (e.g., Pohjolainen et al. 2018).

Further, our analysis showed (RQ3) higher subjective energy poverty to associate with less supportive attitudes toward all our climate measures. These findings indicate that experience of higher energy poverty is associated both with measures that certainly raise energy costs and measures on which the costs can be avoided. Although the strength of these associations decreased after accounting for sociodemographic and party preference characteristics, they remained statistically significant, indicating that the association is robust, i.e., not solely attributable to differences in factors such as party preference or subjective and objective income levels. The results can be seen as being in line with a study conducted in Greece, which found that higher subjective energy poverty reduced the willingness to pay for climate change mitigation (Sarafidis et al. 2024). Our theoretical analysis indicated psychological, social and economic ethically noteworthy risks resulting from climate nudges to people in energy poverty. If the respondents suffering subjective energy poverty recognize these risks, it can partly explain the association between higher subjective energy poverty and lower support toward the measures on which the costs can be avoided.

The persistent statistical link between subjective energy poverty and all dependent variables—even after accounting for socioeconomic factors—indicates that energy poverty independently shapes attitudes toward climate change mitigation, beyond general economic hardship. This effect is particularly strong in the case of fossil fuel taxation.

It seems that subjective energy poverty has an independent association with attitudes toward certain climate change mitigation measures, especially fossil fuel taxation, and thus researchers and decision makers could pay more attention to subjective energy poverty as regards formation of climate policy attitudes. Breaking the trade-offs between climate change mitigation measures and alleviation of subjective energy poverty could help to gain more support for climate policies among citizens. Taking subjective energy poverty into account does not, however, imply that objective poverty is meaningless. Quite contrary, results of our theoretical analysis imply that attention should be paid also to those who are in objective, but not in subjective, energy poverty as they may be especially vulnerable to economic harms from climate nudges.

Combined our results indicate the importance of recognizing subjective and objective energy poverty in decision-making over climate change mitigation measures. Choice of targets, for example, is important as some people do not opt out from measures that harm them whereas others opt out even though they (for climate reasons) should not. Thus, first, energy defaults might be targeted to the better-off individuals. In practice this might mean directing the green defaults mainly on areas where better-off people live and especially not applying them on areas of where less privileged people live. A second solution is to pair climate nudges with financial subsidies. That would diminish both objective and subjective energy poverty and, thus, also diminish both leaving behind and outright harms. A third option is to make the opt-out more salient and clearer (which might, however, make the nudges less effective) (Sunstein 2016, 179). Finally, at least some leaving behind harms could be diminished by complementing defaults with other economically less challenging possibilities for climate actions.

Unlike objective measures, which might rely on specific income thresholds or energy consumption metrics, subjective energy poverty captures how energy scarcity is experienced in daily life. This personal perspective can often reveal more about the real-world impacts of energy insufficiency, as it takes into account factors that objective measures may overlook.

Subjective energy poverty may be a more accurate indicator of energy-related challenges in people’s lives than traditional objective measures. This is because it encompasses the lived experiences of individuals, including their feelings of stress or inadequacy in meeting energy needs. As such, subjective energy poverty can also be a significant predictor of attitudes toward energy policies and expenses. People experiencing this form of poverty might feel more reluctant to support financial measures aimed at addressing broader energy or environmental issues, regardless of their actual income level. From the point of view of our theoretical analysis, there are good reasons for this attitude as both mere subjective energy poverty and mere objective energy poverty (as well as the two together) are risk factors for ethically noteworthy harms from climate nudges. This broadens the understanding of energy poverty and highlights the importance of addressing it through more tailored and sensitive policies that consider both objective and subjective dimensions.

When it comes to causality between subjective energy poverty and attitudes on climate change mitigation measures, we find it more credible that experience of energy poverty precedes attitudes in question. For example, one’s attitudes about fossil fuel taxation might take a more pessimistic turn, if one’s energy poverty situation is getting worse. However, with our cross-sectional data we cannot provide evidence regarding the possible causality in question. To get more information about this, time series and panel data would provide a more comprehensive picture about the phenomenon.

It is crucial to note that fossil fuel taxation and climate nudges should not be considered as equivalent tools in climate policy. Taxation applies broadly across society, while nudges are more targeted interventions whose overall impact on reducing emissions is likely significantly weaker than what could be achieved by increasing fossil fuel taxes. Choice of climate change mitigation measures should be based on thorough analysis weighing and comparing potential harms and benefits of all alternatives, as well as ways to compensate potential harms.

There are also some limitations in our study. The main limitation of the theoretical analysis is that it spells out possibilities and does not provide any information regarding how common the *leaving behind* and *harming outright* harms actually are. Our empirical data include only one time point, and future research would benefit from longitudinal designs to examine how changes in subjective energy poverty over time shape attitudes toward climate policies. Another limitation concerns the conceptualization of subjective energy poverty, which is defined in multiple ways across literature. It is also possible that individuals in certain socioeconomic positions both oppose climate policies and spend a higher share of their income on energy. Although demographic controls were included, they may not fully capture this heterogeneity, and this alternative explanation cannot be ruled out. While this article does not focus on the role of sociodemographic factors in shaping climate attitudes, partly because these relationships have been extensively documented, we acknowledge that such factors may play an equal or even greater role than subjective energy poverty. Future studies could more precisely measure the different dimensions of energy poverty and investigate how each relates to climate policy attitudes. They could also examine whether subjective and objective energy poverty differ in their associations with climate policy support and, if so, how these differences manifest.

Based on our results, we conclude that mitigating energy poverty could provide a win–win situation: as energy poverty and its experience decreases, attitudes toward cost-incurring climate measures may become more positive. This suggests that addressing energy poverty might be a functioning strategy for enhancing public support for essential climate policies. By reducing the immediate economic pressures on households, we can create a more favorable environment for implementing policies aimed at reducing greenhouse gas emissions.

## Supplementary Information

Below is the link to the electronic supplementary material.

Supplementary file1 (PDF 134 KB)

## Data Availability

The data are not yet publicly available but will be made available in the Finnish Social Science Data Archive (fsd.tuni.fi).
