# Supplementary material for: Subjective energy poverty and attitudes on climate change mitigation measures: Empirical and ethical considerations
Source: Ambio. 2026 Jun 28;55(9):2107–18. doi: 10.1007/s13280-026-02434-7 (PMC13437813; doi:10.1007/s13280-026-02434-7)
Supplement: Supplementary file 1 [file 13280_2026_2434_MOESM1_ESM.pdf]

Supplementary Information

Supplementary material has not been peer reviewed.

Title: Subjective energy poverty and attitudes on climate change mitigation measures: Empirical and ethical considerations

Table S1 Descriptive statistics of the dependent variables (scale from 1 to 5).

	**N / %**
**Fossil fuel taxation**<br>(mean=3.07, SD 1.32)	**N=5576**
strongly oppose	16.61%
somewhat oppose	19.46%
neither support or oppose	18.50%
somewhat support	31.29%
strongly support	14.14%
**Renewable energy as default**<br>(mean=3.46, SD=1.17)	N=5568
strongly oppose	7.06%
somewhat oppose	14.34%
neither support or oppose	24.70%
somewhat support	33.62%
strongly support	20.27%
**Low-carbon products most easily available in store**<br>(mean=3.93, SD=1.03)	N=5560
strongly oppose	3.06%
somewhat oppose	4.95%
neither support or oppose	22.97%
somewhat support	33.72%
strongly support	35.30%

Table S2. Descriptive statistics of the independent variables.

	N / %
**Subjective energy poverty** *(I have to compromise on my basic needs in order to pay my energy bills)* (mean=2.54, SD=1.35)	N=5599
Completely disagree	29.20%
Slightly disagree	26.27%
Neither disagree nor agree	17.00%
Slightly agree	16.54%
Completely agree	10.99%
**Subjective financial situation**	N=5536
Bad	11.05%
Moderate	37.21%
Good	51.73%
**Income (logged)** (mean=10.09, SD=0.64)	N=4895
Min	8.11
Max	11.29
**Education level** (mean=2.37, SD=0.65)	N=5626
Primary	9.61%
Secondary	44.17%
Tertiary	46.21%
**Gender** (mean=1.49, SD=0.50)	N=5580
Woman	51.03%
Man	48.97%
**Age group** (mean=1.93, SD=0.80)	N=5840
20–41	35.95%
42–63	34.97%

64–85	29.08%
**Party preference**	N=5517
National Coalition	18.82%
Social Democratic Party of Finland	19.00%
Finns Party	14.61%
Centre Party	8.38%
Green League	11.09%
Left Alliance	7.90%
Other	13.25%
None	6.94%

Table S3 Ordered probit regressions on the associations between subjective energy poverty and attitudes towards different climate change mitigation measures. M1 does not include control variables and M2 includes sociodemographic control variables. Coefficients (robust standard errors in parentheses).

	**Fossil fuel taxation**		**Renewable electricity as default**		**Low-carbon products most easily available in stores**	
	**M1**	**M2**	**M1**	**M2**	**M1**	**M2**
Energy poverty	-0.193*** (-0.0142)	-0.118** (-0.0153)	-0.152*** (-0.014)	-0.0853*** (0.0151)	-0.0986*** (-0.0142)	-0.0346* (0.0152)
Evaluation of own economic situation: Moderate (ref. Poor)		0.0482 (0.0664)		0.144* (0.0650)		0.0656 (0.0662)
Good		0.280*** (0.0704)		0.260***		0.143*
Income (logged)		-0.137*** (0.0344)		-0.100** (0.0333)		-0.0256 (0.0348)
Secondary education (ref. Primary)		-0.120 (0.0613)		0.0243 (0.0607)		-0.0531 (0.0639)
Tertiary education		0.251*** (0.0661)		0.180** (0.0654)		0.125 (0.0684)
Man (ref. Woman)		-0.128*** (0.0355)		-0.266*** (0.0351)		-0.359*** (0.0364)
42-63 (ref. 20-41)		0.0293 (0.0447)		0.0119 (0.0456)		0.0677 (0.0461)
64-85		0.171*** (0.0452)		-0.00249 (0.0457)		0.0519 (0.0465)

Social Democrats (ref. National Coalition Party)		0.464***<br>(0.0537)		0.301***<br>(0.0549)		0.343***<br>(0.0569)
The Finns Party		-0.766***<br>(0.0662)		-0.498***<br>(0.0655)		-0.387***<br>(0.0646)
Centre Party		-0.0308<br>(0.0639)		-0.0201<br>(0.0653)		-0.0238<br>(0.0666)
Green Alliance		1.207***<br>(0.0681)		1.009***<br>(0.0685)		0.899***<br>(0.0737)
Left Alliance		1.033***<br>(0.0798)		0.765***<br>(0.0796)		0.805***<br>(0.0858)
Other		-0.0244<br>(0.0645)		-0.0235<br>(0.0636)		-0.117<br>(0.0643)
None		-0.163*<br>(0.0826)		-0.288***<br>(0.0818)		-0.163*<br>(0.0810)
cut1	-1.484***<br>(-0.0451)	-2.482***<br>(0.338)	-1.890***<br>(-0.0511)	-2.630***<br>(0.330)	-2.062***<br>(-0.0547)	-2.224***<br>(0.345)
cut2	-0.835***<br>(-0.0419)	-1.691***<br>(0.338)	-1.193***<br>(-0.0443)	-1.849***<br>(0.329)	-1.595***<br>(-0.0484)	-1.708***<br>(0.343)
cut3	-0.388***<br>(-0.0407)	-1.150***<br>(0.338)	-0.499***<br>(-0.0414)	-1.069**<br>(0.330)	-0.827***<br>(-0.0431)	-0.857*<br>(0.343)
cut4	0.592***<br>(-0.0416)	0.0195<br>(0.339)	0.423***<br>(-0.0411)	-0.0294<br>(0.330)	0.0447<br>(-0.0413)	0.115<br>(0.343)
N	4450	4450	4452	4452	4455	4455
adj. R-sq	0.0188	0.123	0.0124	0.0825	0.0056	0.0738

* p<0.05, ** p<0.01, *** p<0.001
